# P352: Evaluation of professional practices in hospitals: creation of indicators of quality of care and patient safety

**DOI:** 10.1186/2047-2994-2-S1-P352

**Published:** 2013-06-20

**Authors:** F Toudeft, A Ziri, MK Graba, F Issiakhem, F Saidi, A Makhloufi

**Affiliations:** 1 CHU de Tizi-Ouzou , Tizi-Ouzou, Algeria

## Introduction

In Algeria, improving the quality of care is a priority since 2005, and the impact of actions was felt by patients and caregivers so subjective and varies from one institution to another, because no analysis rational and no research on assessment tools do exist nationwide. CHU of Tizi-Ouzou is one of the pilot sites for the implementation of these indicators.

## Objectives

1 - To determine the incidence of adverse events leading to adverse effects for patients; 2 - Evaluate the degree of patient satisfaction with regard to various hospital services; 3 - Find the underlying causes of iatrogenesis; 4 - Define a strategy to improve the quality of care.

## Methods

THERE are two surveys, one descriptive cross-sectional, followed by a prospective, analytical type, with patients hospitalized for a minimum of 24 to 48 hours over a period of two years.

## Results

Preliminary results showed that of 656 patients hospitalized for an average stay of 7 days, the incidence of adverse events was 16.5%, the confirmation rate is 87.96%. The event most frequently encountered in the first passage is nosocomial infection (11.1%) in the second passage, unwanted physical harm (10.2%) in the third passage, nosocomial infection (3.7%).

As the survey of patient satisfaction, weaknesses in the quality of care in our institution are by far the advice given to the patient's discharge (57.8%), the information given by the doctor (57.01% ), and the identification of the function of the staff (49.5%).

## Conclusion

Despite the constraints, this study allows us to better know the quality of care at our institution and the procedure to be adopted to establish the assessment tools, however, the implementation of a quality project requires a system efficient information in order to analyze a priori and a posteriori risk inherent in each activity or process of care measures withdrawal, reduction and / or recovery and a battery of indicators applicable to our care facilities and generalizable at regional or national level.

## Disclosure of interest

None declared

